# Comparison between relative and absolute quantitative real-time PCR applied to single-cell analyses: Transcriptional levels in a key neuron for long-term memory in the pond snail

**DOI:** 10.1371/journal.pone.0279017

**Published:** 2022-12-12

**Authors:** Dai Hatakeyama, Nozomi Chikamoto, Kanta Fujimoto, Takashi Kitahashi, Etsuro Ito

**Affiliations:** 1 Faculty of Pharmaceutical Sciences, Tokushima Bunri University, Tokushima City, Japan; 2 Department of Biology, Waseda University, Tokyo, Japan; 3 Kushiro Nature Conservation Office, Ministry of the Environment Government of Japan, Kushiro City, Japan; 4 Graduate Institute of Medicine, School of Medicine, Kaohsiung Medical University, Kaohsiung City, Taiwan; University of Montreal, CANADA

## Abstract

Quantitative real-time PCR (qPCR) is a powerful method for measuring nucleic acid levels and quantifying mRNA levels, even in single cells. In the present study, we compared the results of single-cell qPCR obtained by different quantification methods (relative and absolute) and different reverse transcription methods. In the experiments, we focused on the cerebral giant cell (CGC), a key neuron required for the acquisition of conditioned taste aversion in the pond snail *Lymnaea stagnalis*, and examined changes in the mRNA levels of 3 memory-related genes, cAMP-response element binding proteins (LymCREB1 and LymCREB2) and CREB-binding protein (LymCBP), during memory formation. The results obtained by relative quantification showed similar patterns for the 3 genes. For absolute quantification, reverse transcription was performed using 2 different methods: a mixture of oligo d(T) primers and random primers (RT method 1); and gene-specific primers (RT method 2). These methods yielded different results and did not show consistent changes related to conditioning. The mRNA levels in the samples prepared by RT method 2 were up to 3.3 times higher than those in samples prepared by RT method 1. These results suggest that for qPCR of single neurons, the efficacy and validity do not differ between relative and absolute quantification methods, but the reverse transcription step critically influences the results of mRNA quantification.

## Introduction

Quantitative real-time PCR (qPCR) is routinely used for detecting and measuring levels of nucleic acids, such as mRNA [1–3], viral RNA [4–6], bacterial DNA and RNA [7, 8], and degraded single-stranded DNA [9]. For measuring RNA levels, RNA is first reverse-transcribed into cDNA, and then the synthesized cDNA is amplified using target-specific primers in the PCR reaction. During the PCR process, the amount of the PCR product is monitored as fluorescence intensity in real time using non-specific double-stranded DNA-binding dyes or sequence-specific hybridization probes [10]. Non-specific double-stranded DNA-binding dyes include SYBR Green, SYTO9 [11], EvaGreen [12], and BrightGreen [13, 14]. Dual-labeled hybridization probes (such as the TaqMan^®^ probe) have several advantages as sequence-specific hybridization probes, particularly with regard to specificity [10].

On the basis of real-time monitoring of fluorescence intensity, the expression of a target gene can be measured by relative quantification or absolute quantification. In relative quantification, changes in gene expression are expressed as fold-change compared with the control sample. In relative quantification, the comparative threshold method, in which the Ct value of a gene of interest is compared with that of a reference gene, is used to normalize the gene expression in each sample (the 2^-ΔΔCt^ value) [15]. Absolute quantification requires a standard curve obtained by a series of known copies of the target molecule [16]. Quantification of the copy numbers of a target molecule in unknown samples is accomplished by measuring the threshold cycle and determining the starting copy number on the basis of a standard curve.

In studies of memory consolidation in the pond snail *Lymnaea stagnalis*, we have utilized qPCR to quantify mRNA levels of even a single cell [17, 18]. *Lymnaea stagnalis* can learn conditioned taste aversion (CTA) and consolidate it into long-term memory [19–22]. For long-term memory consolidation, a pair of cerebral giant cells (CGCs) function as key neurons [23, 24]. The CGCs are large enough (up to 150 μm in diameter) to perform electrophysiologic experiments. Injection of cyclic AMP (cAMP) into the CGCs increases the amplitude of excitatory postsynaptic potentials in postsynaptic B1 and B3 cells [25, 26], suggesting that cAMP-responsive element-binding protein (CREB) plays an important role in the CGCs during memory consolidation. Sadamoto et al. successfully cloned the cDNAs for 2 homologues of CREB (LymCREB1 and LymCREB2) from the central nervous system (CNS) of *Lymnaea* [27] and demonstrated their colocalization in the CGCs [27, 28]. The mRNA of CREB-binding protein (CBP), a coactivator of CREB [29], was also found to colocalize with the mRNA of LymCREB1 and LymCREB2 in the CGCs [30]. We were inspired to perform single-cell qPCR on the CGCs as these neurons are easily identified and surgically isolated under a stereoscopic microscope with glass electrodes and super-fine-tip forceps [17, 18].

In the present study, we trained snails using a CTA training protocol and examined changes in the mRNA levels of LymCREB1, LymCREB2, and LymCBP in the CGCs isolated from the CNS. For qPCR, we compared the results of both relative quantification and absolute quantification. To further improve the single-cell qPCR technique, we compared 2 different reverse transcription methods for absolute quantification.

## Materials and methods

### Snails

*Lymnaea stagnalis* with an 18–25 mm shell length obtained from our snail-rearing facility (original stocks from Vrije Universiteit Amsterdam) were used. The snails were fed turnip leaves (*Brassica rapa var*. *peruviridis*, known as Komatsuna in Japanese) *ad libitum* and maintained in dechlorinated tap water (i.e., pond water) under a 12 h light:12 h dark cycle at 20°C–23°C.

### Conditioned taste aversion paradigm

**Fig 1:**
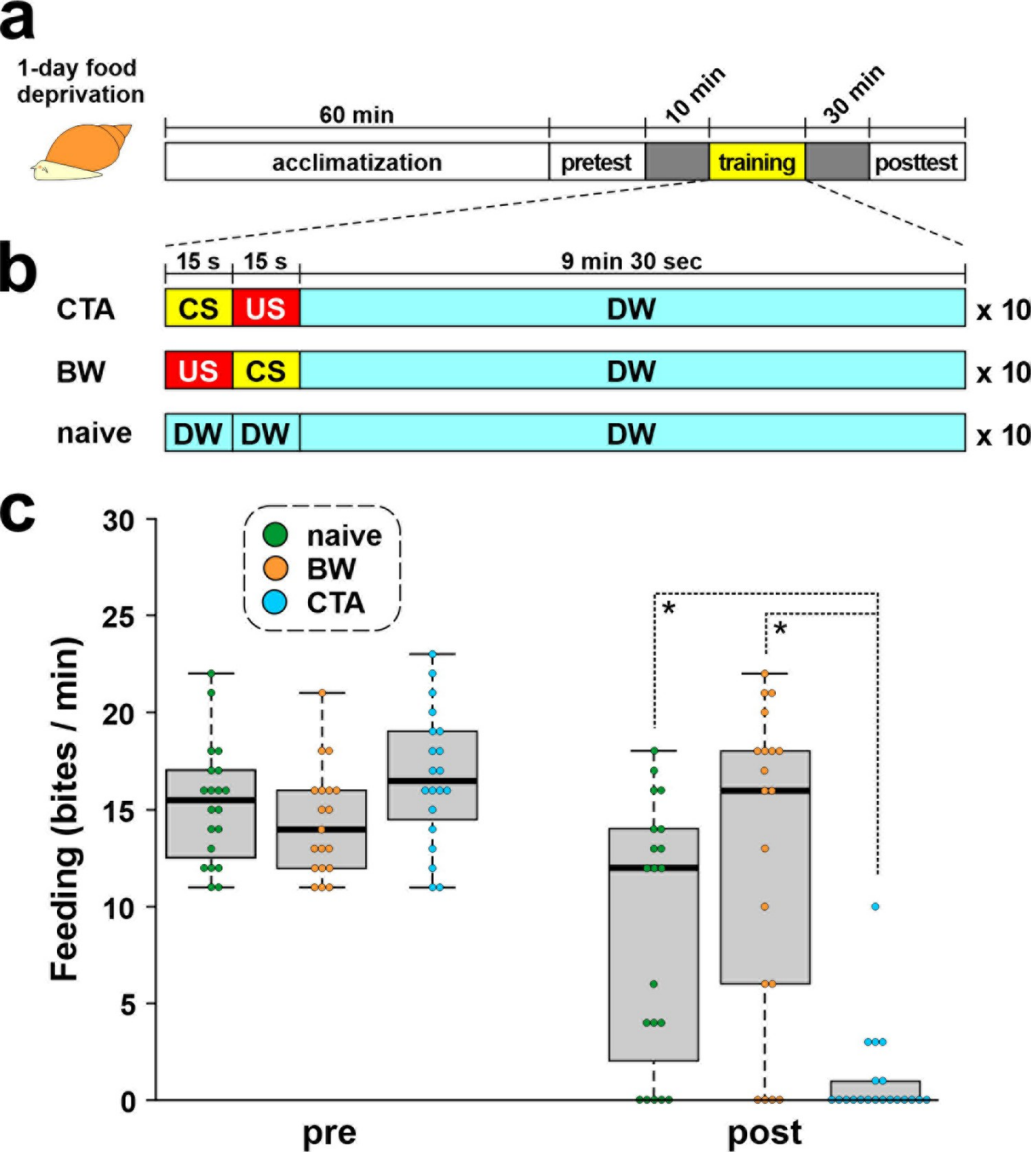
Conditioned taste aversion (CTA) training for *Lymnaea*. (A) Diagram of the total procedure of CTA. (B) Procedures used for CTA training, backward (BW) training, and naïve control. CS: conditioned stimulus (10 mM sucrose solution), US: unconditioned stimulus (10 mM KCl solution). (C) Box-and-whisker plots show the learning and memory scores of CTA (i.e., number of bites to CS) examined at the pretest and posttests. Green, orange, and blue dots show naïve-control, BW-trained, and CTA-trained snails, respectively. The number of snails was at least 10 in each condition. *p < 0.05 by Holm’s post-hoc test.

To obtain good behavioral training scores, the snails were not fed for 1 day prior to the behavioral experiment [14, 31]. The conditioning strategies are summarized in Fig 1A and 1B [32, 33]. The pretest, training, and posttest were performed in a polystyrene petri dish (diameter 35 mm) [34]. All snails were acclimatized in distilled water (DW) for 1 h and given a pretest (Fig 1A). In the pretest, 2-mL of a 10-mM sucrose solution was applied to the lips of the snail for 15 s as a conditioned stimulus (CS), and the number of feeding responses (i.e., rasping movements of the buccal mass) in DW was counted for 1 min. CTA learning in *Lymnaea* was induced by pairing a 10-mM sucrose solution as the CS and a 10-mM KCl solution as an unconditioned stimulus (US) (Fig 1B). The duration of both the CS and US was 15 s, with a 15-s interstimulus interval between the onset of the CS and the onset of the US. Snails received 10 paired CS-US trials with 10-min intertrial intervals.

To validate associative learning, backward (BW)-training (i.e., US-CS) and naïve-control groups were included. For the naïve-control group, DW was applied to the lips instead of the CS or US. In the posttest session, snails were again challenged with the CS, and the number of bites was recorded for 1 min after a 15-s application of the CS. All behavioral experiments were performed in the morning [35], and the posttests were performed with investigators blind to the group condition.

### Isolation of single CGCs

The *Lymnaea* CNS was isolated between 30 min and 4 h after the posttest in *Lymnaea* saline (50 mM NaCl, 1.6 mM KCl, 10 mM HEPES-NaOH [pH 8.1], 2.0 mM MgCl_2_, and 3.5 mM CaCl_2_). The CGCs were then isolated in high osmolality medium (Leibovitz’s L-15 medium [Gibco BRL, Gaithersburg, MD, USA] and Saline-S). Isolated cells were transferred into a CGC lysis solution (0.45 μL DEPC-treated water [Thermo Fisher Scientific, Waltham, MA, USA], 0.25 μL RNase inhibitor [Applied Biosystems, Foster City, CA, USA]), frozen in liquid nitrogen, and stored at -80°C.

### Cell lysis and reverse transcription

Two methods of reverse transcription (RT) were used.

[RT Method 1] After the cell was lysed by incubating the tube at 65°C for 5 min, cDNA synthesis was performed with the mixture of oligo d(T) primers and random primers using ReverTra Ace^®^ qPCR RT Master Mix with gDNA Remover (Toyobo, Osaka, Japan) following the manufacturer’s protocol.

**Fig 2:**
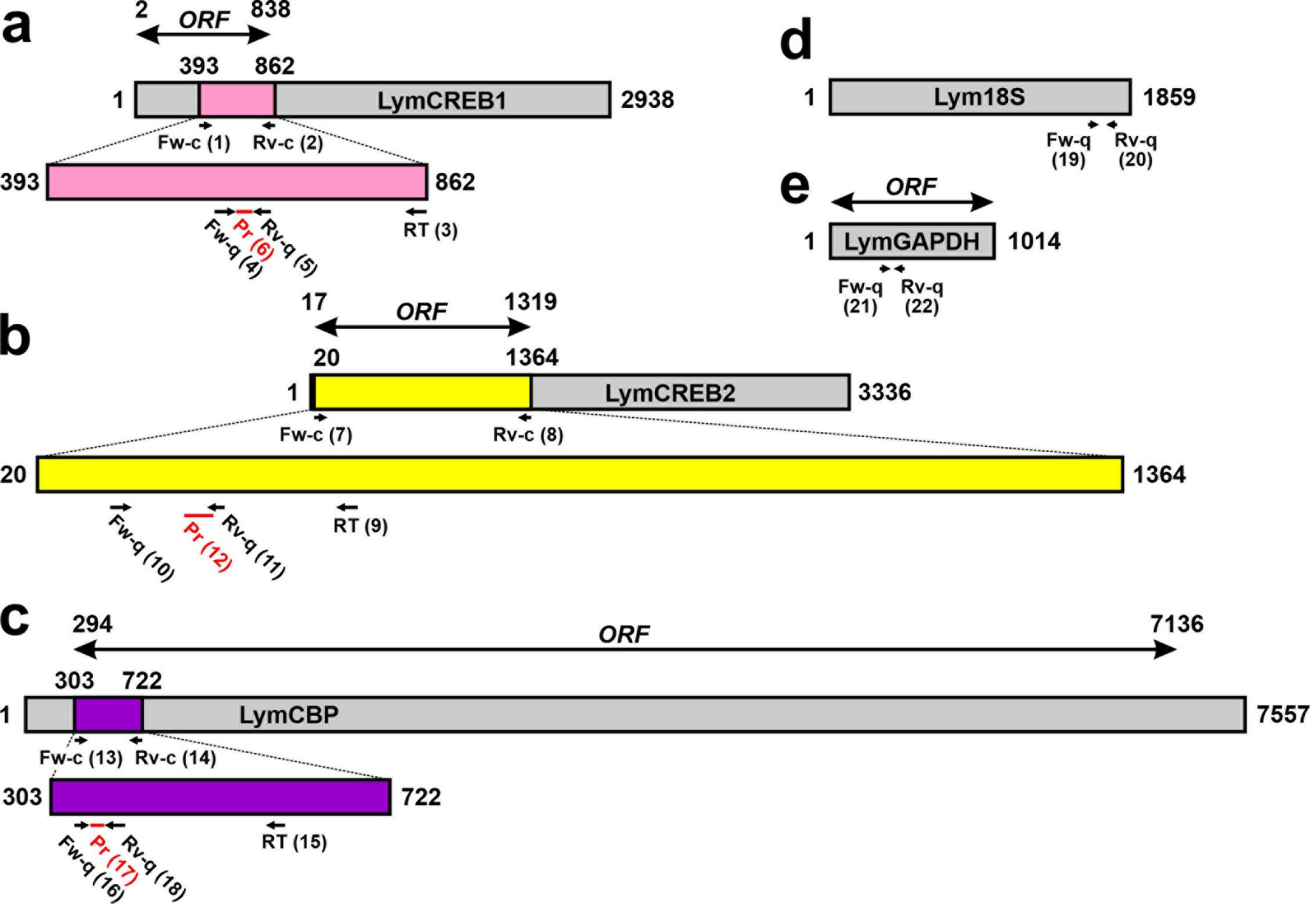
Positions of primers and probes used in the experiments. (A) LymCREB1 [GenBank Accession No.: AB041522], (B) LymCREB2 [AB083656], (C) LymCBP [AB217914], (D) Lym18S [Y09018], and (E) LymGAPDH [MH687363]. Fw-c: forward primer for cloning, Rv-c: reverse primer for cloning, RT: gene-specific primer for reverse transcription, Fw-q: forward primer for qPCR, Rv-q: reverse primer for qPCR, Pr: TaqMan probe. Numbers in parentheses indicate the corresponding primers and probes shown in Table 1. Numbers without parentheses indicate nucleotide numbers. ORF: open reading frame.

[RT Method 2] The cell lysis and reverse transcription procedures were performed as previously described with modifications [17, 18, 36]. First, a CGC was mixed with 4.45 μL of a denaturing buffer containing gene-specific RT primers (20 ng/μL yeast tRNA, 0.2 μM each gene-specific primer for LymCREB1, LymCREB2, and LymCBP, 0.1% NP40, and 0.3 unit/mL Prime RNase inhibitor [Eppendorf, Hamburg, Germany]), and then treated at 65°C for 1 min to break down the cell membrane. The positions and nucleotide sequences of the gene-specific primers for reverse transcription are summarized in Fig 2 and Table 1 [No. (3) for LymCREB1, No. (9) for LymCREB2, and No. (15) for LymCBP]. Immediately after the denaturing step, 5.55 μL of RT buffer (1.80×PCR Buffer II [Applied Biosystems], 9.90 mM MgCl_2_, 0.90 mM dNTP mix, Prime RNase inhibitor, and 0.90 units/ml MultiScribe^TM^ Reverse Transcriptase [Applied Biosystems]) was added to the tube, and reverse transcription was performed (incubation at 25°C for 10 min, 48°C for 30 min, and at 95°C for 5 min). All RT samples were then diluted 1:5 by adding 40 μL of DEPC-treated water.

**Table 1:** Nucleotide sequences of primers and probes used in the experiments.

No.	Primer/probe name	Nucleotide sequence
**LymCREB1**		
(1)	cloning_Fw (Fw-c)	5’-GAA CAT TGC AGC TGA CAG CAC ATG GAG ATG-3’
(2)	cloning_Rv (Rv-c)	5’-AAA AGG AAC ACT GAT AAT CAG TCA TGC ATC-3’
(3)	RT	5’-AGG AAC ACT GAT AAT CAG TCA TGC ATC-3’
(4)	qPCR_Fw (Fw-q)	5’-GCT CAA GGT GTT GTT ATG ACA GGT-3’
(5)	qPCR_Rv (Rv-q)	5’-TTC GAG AGC CTT CTT CAG ACA TG-3’
(6)	qPCR_probe (Pr)	5’- FAM -TCC CAT ATC TTC CCC ACA AC- TAMRA -3’
**LymCREB2**		
(7)	cloning_Fw (Fw-c)	5’-GCT AAA TGA CAA TCA TGT CAG CTT AAA TTG-3’
(8)	cloning_Rv (Rv-c)	5’-GAA TCC CAG TTC ATT AAT GCA CTA TTT GAC-3’
(9)	RT	5’-CAA GAA ATT TTC AAT ATC CTT GGA TGG-3’
(10)	qPCR_Fw (Fw-q)	5’-CCT AGC TAC GGC TGC TAT ATC TAC AAA-3’
(11)	qPCR_Rv (Rv-q)	5’-GTC AAC AAG TCC AGG TCC CAT T-3’
(12)	qPCR_probe (Pr)	5’- FAM -CTG CCA AGC AGC AAA TCT TCG TTC CA- TAMRA -3’
**LymCBP**		
(13)	cloning_Fw (Fw-c)	5’-GAT GTA AAC ATG GCC GAC CAC CAA CTT G-3’
(14)	cloning_Rv (Rv-c)	5’-AGA CGA TGT AGC AAC TTT GTT TGT ATT GGC-3’
(15)	RT	5’-TGT TAA GAG ACA TCG GCA TTG ATG-3’
(16)	qPCR_Fw (Fw-q)	5’-GCC CTC CGG CCA ACA AGA A-3’
(17)	qPCR_Rv (Rv-q)	5’-TAT TAT CGC TGG GTG TAT TGA GAG AT-3’
(18)	qPCR_probe (Pr)	5’- FAM -ACC CAG AAT CGG ATC AC- TAMRA -3’
**Lym18S**		
(19)	qPCR_Fw (Fw-q)	5’-CTC CTT CGT GCT AGG GAT TG-3’
(20)	qPCR_Rv (Rv-q)	5’-GTA CAA AGG GCA GGG ACG TA-3’
**LymGAPDH**		
(21)	qPCR_Fw (Fw-q)	5’-CAA CAA CCG ACA AAG CAA-3’
(22)	qPCR_Rv (Rv-q)	5’-CAT AAC AAA CAT AGG GGC A-3’

### Relative qPCR

Real-time PCR was performed in a 10-μL mixture containing 3 μL of 10-fold diluted cDNA solution and 0.3 μM each of the forward and reverse primers using BrightGreen 5×qPCR MasterMix-ROX (Applied Biological Materials Inc., Richmond, BC, Canada). The PCR primers are indicated as “Fw-q” and “Rv-q” in Fig 2 and Table 1. The reaction was carried out at 95°C for 20 s, followed by 40 cycles at 95°C for 3 s and at 60°C for 30 s with StepOnePlus^TM^ (Applied Biosystems). A melting curve analysis was carried out between 60°C and 95°C, with a plate read every 0.3°C. As reference genes, 18S ribosomal RNA (Lym18S; GenBank Accession No.: Y09018) and glyceraldehyde 3-phosphate dehydrogenase (GAPDH) mRNA (LymGAPDH; GenBank Accession No.: MH687363) were used [37, 38]. The positions and nucleotide sequences of the PCR primers are summarized in Fig 2 and Table 1 [No. (19) and (20) for Lym18S, and No. (21) and (22) for LymGAPDH].

### Absolute qPCR

To produce the standard DNAs, partial cDNAs of LymCREB1 (GenBank Accession No.: AB041522), LymCREB2 (GenBank Accession No.: AB083656), and LymCBP (GenBank Accession No.: AB217914) were obtained by standard RT-PCR. The positions and nucleotide sequences of the cloning primers are summarized in Fig 2 and Table 1 [No. (1) and (2) for LymCREB1, No. (7) and (8) for LymCREB2, and No. (13) and (14) for LymCBP]. The PCR products were then cloned into a pGEM^®^-T Easy Vector (Promega, Madison, WI, USA). Using the plasmids as a template, standard RNAs were synthesized using a MAXIscript^TM^ T7 Transcription Kit (Invitrogen, Waltham, MA, USA) and purified with an RNeasy^®^ Mini Kit (QIAGEN). To prepare the standard cDNA, serially diluted standard RNAs (5×10^1^–5×10^6^ copies/μL) were reverse-transcribed in 10 μL of reaction mixture using 0.90 units/mL MultiScribe^TM^ Reverse Transcriptase (Applied Biosystems) with 2.5 μM of gene-specific RT primers, whose positions and nucleotide sequences are summarized in Fig 2 and Table 1 [No. (3) for LymCREB1, No. (9) for LymCREB2, and No. (15) for LymCBP], and diluted 1:5.

The diluted RT solution of samples and standards (1 μl) were added to a PCR-reaction mixture (final concentration: 1×TaqMan® Gene Expression Master Mix [Applied Biosystems], 50 nM each forward and reverse primer, and 50 nM probe). The positions and nucleotide sequences of primers and probes are summarized in Fig 2 and Table 1 [No. (4) to (6) for LymCREB1, No. (10) to (12) for LymCREB2, and No. (16) to (18) for LymCBP]. The reaction was carried out at 95°C for 10 min, followed by 50 cycles of 95°C for 15 s and at 60°C for 1 min with StepOnePlus^TM^. In the assay, several doses of standard cDNA (1×10^1^–1×10^6^ copies) were applied in triplicate to estimate the inter-assay coefficients of variation between runs.

### Statistics

All pPCR data were analyzed with Origin 6.0 statistical software and are shown as bar graphs with mean ± SEM. A 1-way analysis of variance (ANOVA) followed by Scheffé’s or Tukey’s post-hoc tests, and 2-way ANOVA followed by Holm’s post-hoc tests were used for comparison among groups. Linear regression analyses were performed using Microsoft Excel 2016. Behavioral analyses and box plots were performed using computer software R (version 3.6.0; https://www.r-project.org/). Parallel tests of regression lines were performed using IBM SPSS Statistics software (version 28).

## Results

### Memory consolidation of CTA

Conditioned (CTA-trained) snails, naïve-control, and BW-trained snails were prepared (Fig 1). Without conditioning, a CS induces a large number of bites and a US evokes a withdrawal response [20]. The CTA group exhibited a significantly lower feeding response to sucrose compared with the BW group or the naïve-control group (Fig 1C; *p* < 0.001 by 2-way ANOVA, *p* < 0.05 by Holm’s post-hoc test), indicating that CTA was established. The CNS was then dissected from these snails, and the CGCs were isolated from the CNS for single-cell qPCR.

### Relative quantification

**Fig 3:**
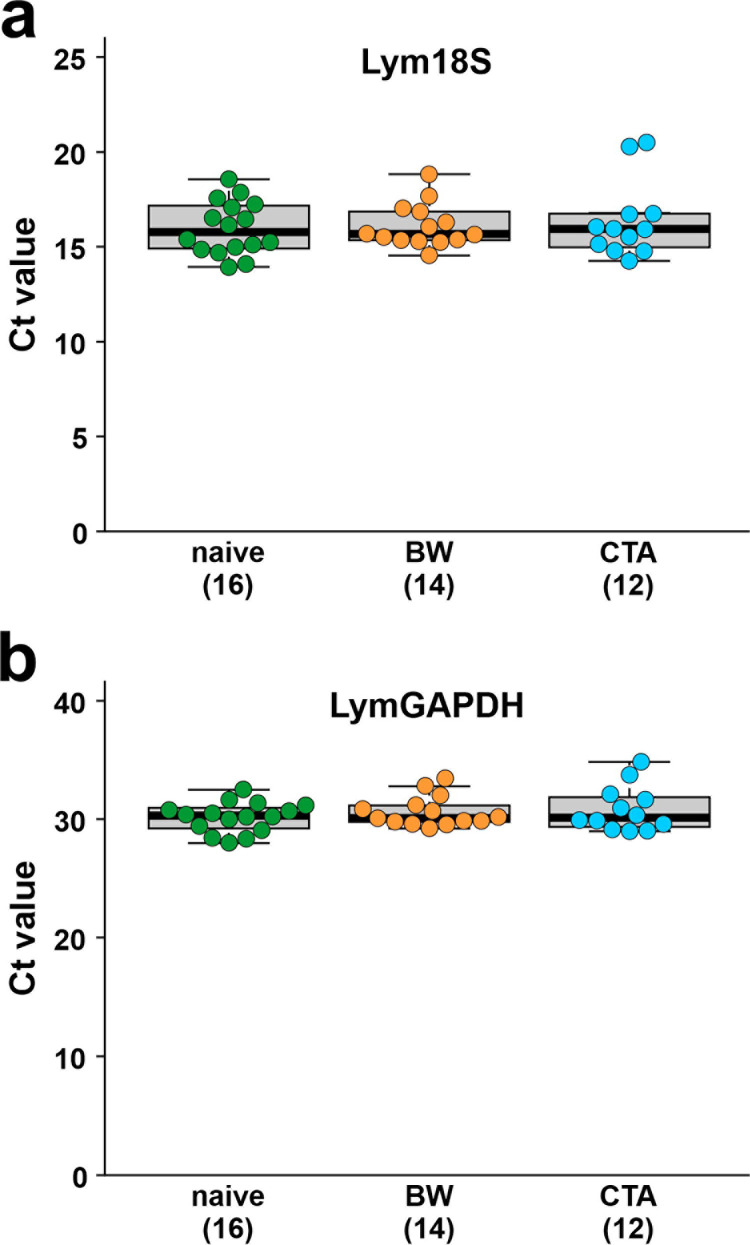
Variation in Ct values of the 2 reference genes used for relative quantification. (A) Lym18S, and (B) LymGAPDH. Green, orange, and blue dots show naïve-control (*n* = 16), BW-trained (*n* = 14), and CTA-trained snails (*n* = 12), respectively. Data are expressed as mean ± SEM.

For relative quantification of the mRNA levels of LymCREB1, LymCREB2, and LymCBP, reverse transcription of isolated CGCs was performed with the mixture of oligo d(T) and random primers using ReverTra Ace^®^ qPCR RT Master Mix with gDNA Remover (see RT Method 1 described in the “Materials and methods”). As reference genes, we selected Lym18S and LymGAPDH. A 1-way ANOVA revealed no significant differences in the Ct values of Lym18S (*p* = 0.83) and LymGAPDH (*p* = 0.46) among the 3 different conditioning paradigms (Fig 3), suggesting that these 2 genes were suitable as the reference genes.

**Fig 4:**
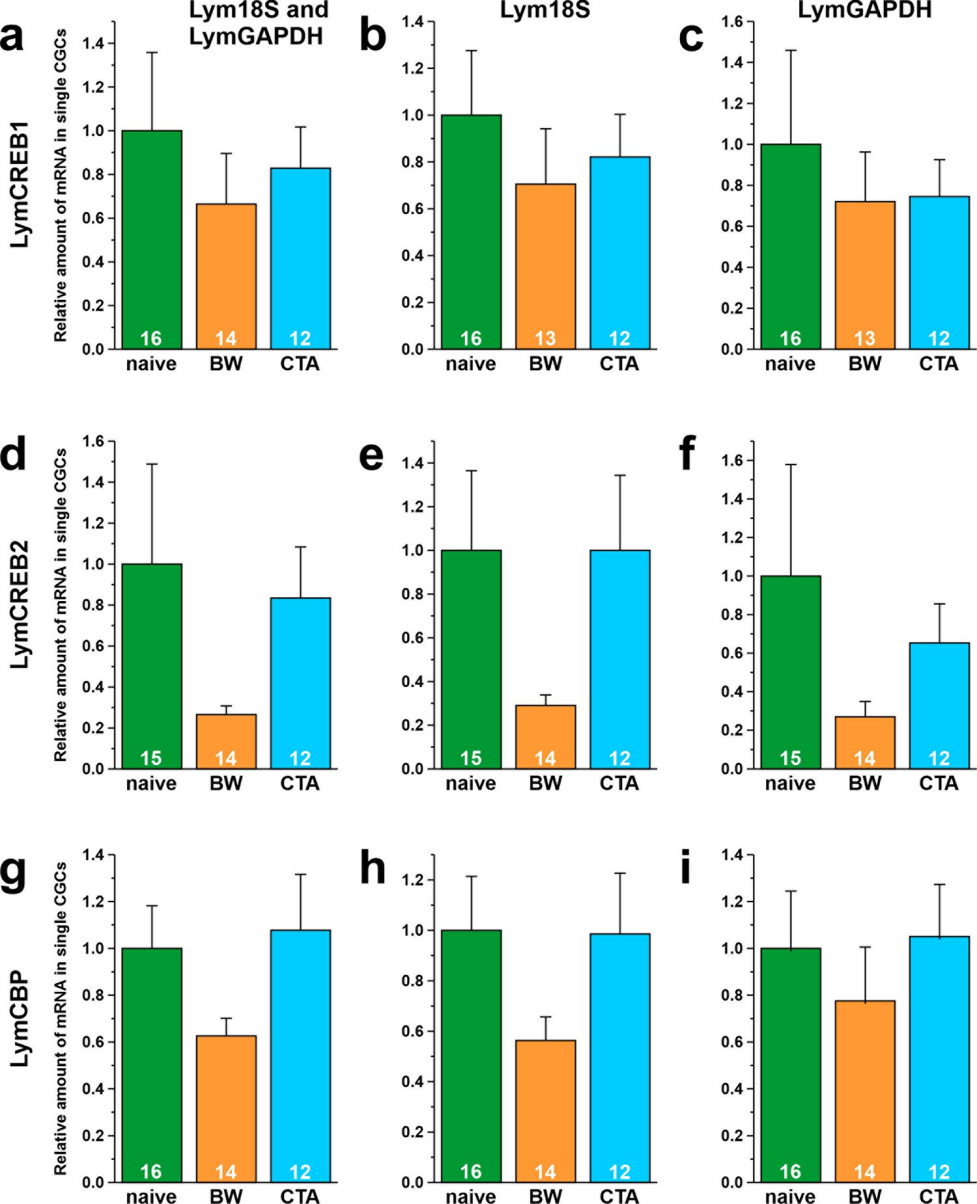
Comparison of reference genes for qPCR. Results of relative quantification of LymCREB1 (A-C), LymCREB2 (D-F), and LymCBP (G-I) mRNA levels. Two reference genes (A, D, G: with Lym18S and LymGAPDH) or a single reference gene (B, E, H: with Lym18S; C, F, I: with LymGAPDH) were used for normalizing the expression levels. Green, orange, and blue bars show naïve-control, BW-trained, and CTA-trained snails, respectively. Data are expressed as mean ± SEM. The number of samples is indicated at the bottom of the bar graph.

The amounts of LymCREB1, LymCREB2, and LymCBP mRNA were normalized with 2 reference genes (both Lym18S and LymGAPDH, Fig 4A, 4D and 4G) or a single reference gene (only Lym18S, Fig 4B, 4E and 4H; or only LymGAPDH, Fig 4C, 4F and 4I). For LymCREB1, although there were no significant differences in the mRNA levels among the 3 conditioning protocols, the mRNA levels tended to be the highest in the naïve-control snails and lower in the BW- and CTA-trained snails (Fig 4A–4C). The relative amounts of LymCREB1 mRNA in the BW-trained and CTA-trained snails differed, however, depending on the gene(s) used as the reference; CTA was higher than BW when both reference genes were used or only Lym18S was used (Fig 4A and 4B); and BW and CTA were at similar levels when only LymGAPDH was used (Fig 4C). For LymCREB2 (Fig 4D–4F) and LymCBP (Fig 4G–4I), there were no significant differences in the mRNA levels among the 3 conditioning groups, similarly to the case of LymCREB1. The mRNA levels of both genes, however, were lower in the BW-trained snails compared with the naïve-control and CTA-trained snails. Thus, similar patterns of changes in the mRNA levels were observed irrespective of the reference gene used.

### Absolute quantification

For absolute quantification of the mRNA levels of LymCREB1, LymCREB2, and LymCBP, reverse transcription of isolated CGCs was performed by 2 different methods. *RT Method 1*: Samples used for the relative quantification, which were reverse-transcribed with a mixture of oligo d(T) and random primers using ReverTra Ace^®^ qPCR RT Master Mix with gDNA Remover, were also subjected to absolute quantification. *RT Method 2*: In this method, we used MultiScribe^TM^ Reverse Transcriptase (Applied Biosystems) and the gene-specific primers for LymCREB1, LymCREB2, and LymCBP for reverse transcription.

**Fig 5:**
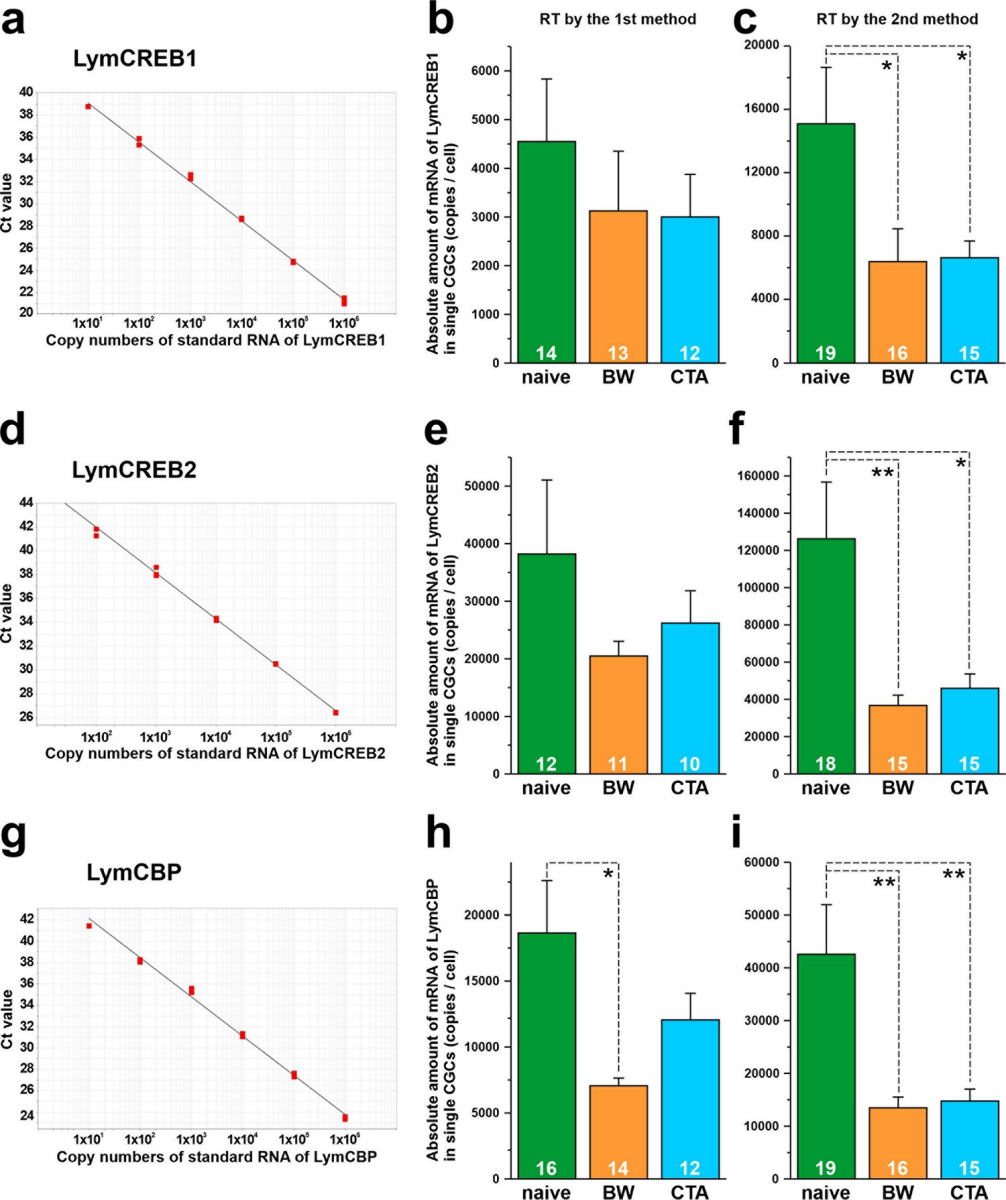
Results of absolute quantification of LymCREB1, LymCREB2 and LymCBP mRNA levels. Standard curves of LymCREB1 (A), LymCREB2 (D), and LymCBP (G). The mRNA levels in the CGCs reverse-transcribed with the mixture of oligo d(T) and random primer (B, E, H) or gene-specific primers (C, F, I). Green, orange, and blue bars show naïve-control, BW-trained, and CTA-trained snails, respectively. Quantification data are expressed as mean ± SEM. The results were analyzed using 1-way ANOVA followed by Tukey’s post-hoc test (*p < 0.05, **p < 0.01). The number of samples is indicated at the bottom of the bar graph.

The standard curve of each gene was drawn as a straight line and covered all unknown samples measured (Fig 5A, 5D and 5G). The samples reverse-transcribed by RT method 1 showed the highest mRNA levels in the naïve-control snails and lower levels in the BW-trained and CTA-trained snails for all 3 target genes (Fig 5B, 5E and 5H). No statistically significant differences, however, were detected among the 3 groups for LymCREB1 (Fig 5B; *p* = 0.574 by 1-way ANOVA) and LymCREB2 (Fig 5E; *p* = 0.340 by 1-way ANOVA). For LymCBP, the naïve-control group showed significantly higher mRNA levels compared with the BW-trained snails (Fig 5H; *p* < 0.05 by 1-way ANOVA; *p < 0.05 by Scheffé’s post-hoc test).

On the other hand, in the samples reverse-transcribed by RT method 2, the mRNA levels of the 3 target genes were highest in the naïve-control snails (Fig 5C, 5F and 5I). The BW and CTA training groups showed significantly lower mRNA levels compared with the naïve-control for LymCREB1 (Fig 5C; *p* < 0.05 by 1-way ANOVA; *p < 0.05 by Scheffé’s post-hoc test), LymCREB2 (Fig 5F; *p* < 0.01 by 1-way ANOVA; *p < 0.05 and **p < 0.01 by Scheffé’s post-hoc test), and for LymCBP (Fig 5I; *p* < 0.01 by 1-way ANOVA; **p < 0.01 by Scheffé’s post-hoc test). Comparison of the results of the 2 RT methods revealed that the mRNA levels in the samples reverse-transcribed by the RT method 2 were 1.2 to 3.3 times higher than those in the samples reverse-transcribed by RT method 1 (Table 2).

**Table 2:** Ratio between average values of mRNA levels (RT method 2/RT method 1) calculated by absolute quantification.

	Naïve	BW	CTA
LymCREB1	3.3	2.0	2.2
LymCREB2	3.3	1.8	1.8
LymCBP	2.3	1.9	1.2

### Examination of affinity and accuracy of PCR by agarose gel electrophoresis

We performed the agarose gel electrophoresis after qRT-PCR to examine the affinity and accuracy of SYBR Green and TaqMan probes used in relative and absolute quantification, respectively. This experiment was individually carried out at 2 different laboratories in Waseda University and Tokushima Bunri University, and indicated that the only single bands of LymCREB1, LymCREB2 and LymCBP were clearly observed in both groups (S1 Fig). In addition, all melting curves showed the single and sharp peaks. These results showed that we can exclude the possibility of forming primer dimers and non-specific amplification.

### Correlation analyses of mRNA levels among LymCREB1, LymCREB2, and LymCBP

**Fig 6:**
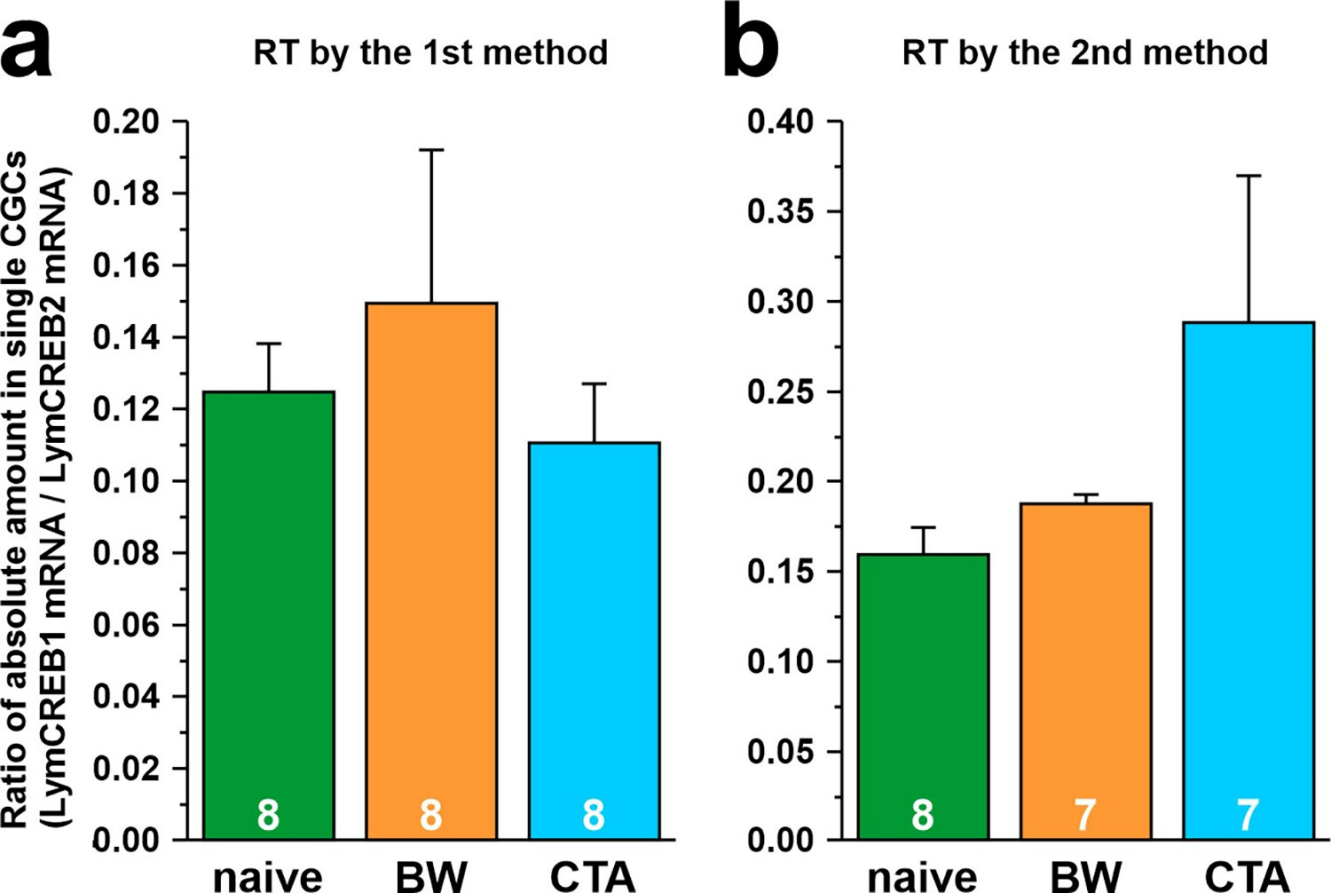
Ratio of LymCREB1 mRNA/LymCREB2 mRNA in a single isolated CGC. The data were obtained by absolute quantification with samples reverse-transcribed by RT method 1 (A) and RT method 2 (B). Green, orange, and blue bars show naïve-control, BW-trained, and CTA-trained snails, respectively. Data are expressed as mean ± SEM. *p*-values of 1-way ANOVA are 0.61 (A) and 0.15 (B). The number of samples is indicated at the bottom of the bar graph.

In another molluscan species, *Aplysia californica*, CREB2 is known to suppress CREB1-mediated transcriptional activity by forming a heterodimer with CREB1 during long-term memory consolidation [39]. Thus, to elucidate the correlation between the mRNA levels of LymCREB1 and LymCREB2, we calculated the ratio of LymCREB1 and LymCREB2 mRNA levels obtained by absolute quantification (Fig 6). Although there were no significant differences among the 3 conditioning groups (Fig 6A, RT method 1, *p* = 0.15; Fig 6B, RT method 2, *p* = 0.61; 1-way ANOVA), the pattern of the graphs differed from one another; the LymCREB1/LymCREB2 ratio was highest in the BW-trained group for RT method 1 and in the CTA-trained group for RT method 2.

**Fig 7:**
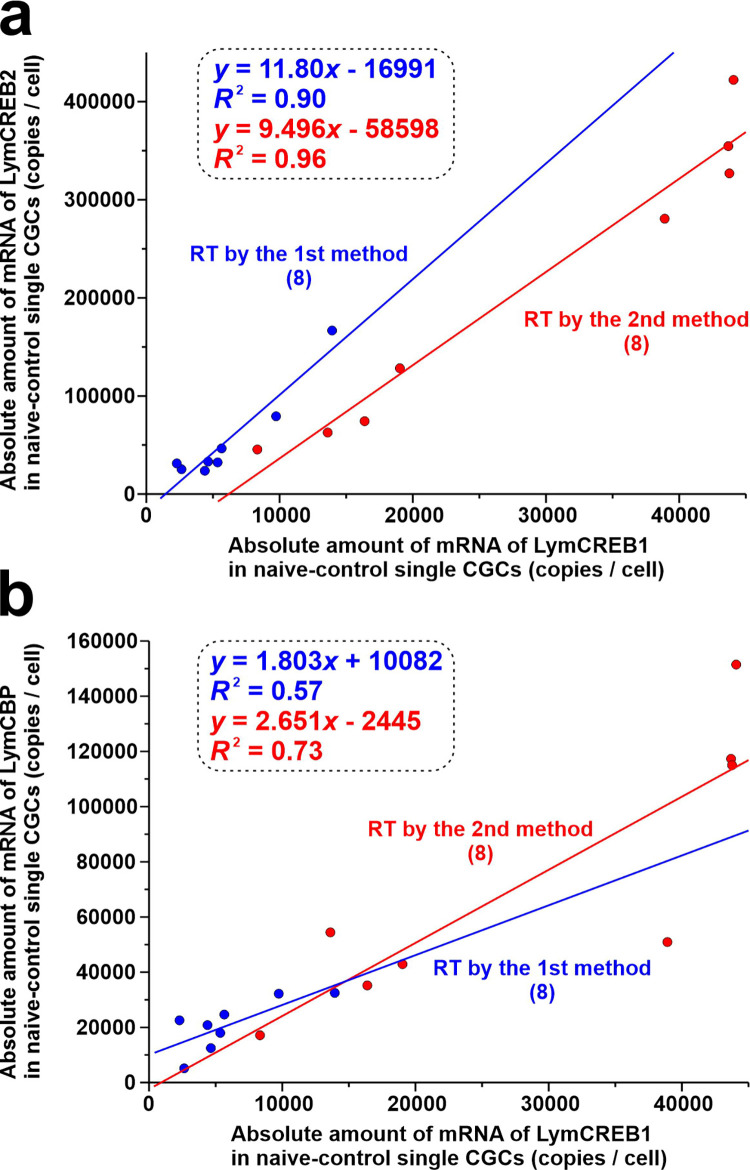
Linear regression analyses to evaluate the relationship between mRNA levels of LymCREB1 (*X* axis) and LymCREB2 [*Y* axis in (A)] and LymCBP [*Y* axis in (B)] calculated by absolute quantification. The red dots and lines indicate samples prepared by RT method 1 and the blue dots and lines indicate samples prepared by RT method 2. The number of samples was 8 for each group. Equation and coefficient of determination (*R*^2^ values) of lines are shown in insets. Slopes and *F*-test results are summarized in Table 3. Statistical analyses suggested that the 2 regression lines are parallel in LymCREB2/LymCREB1 and LymCREB2/LymCBP [*p* = 0.404 (A); *p* = 0.726 (B)].

In *Aplysia*, a related species of *Lymnaea*, CREB1 is reported to interact with both CREB2 and CBP [39–41]. The amino acid sequences of CREB1, CREB2 and CBP of *Aplysia* are highly identical with those of *Lymnaea*, suggesting that LymCREB1 interacts with both LymCREB2 and LymCBP. Here, to compare the mRNA levels of LymCREB1 with LymCREB2, and LymCREB1 with LymCBP, we individually plotted mRNA levels of these genes measured by absolute quantification for each of the naïve-control snails and compared them between the 2 different RT methods (Table 3 and Fig 7). For the ratio of LymCREB1 to LymCREB2, the slopes of the regression lines were around 10 (Table 3 and Fig 7A). The slopes of the 2 regression lines (RT method 1 and RT method 2) did not differ significantly between LymCREB2/LymCREB1 or between LymCREB2/LymCBP [(A) *p* = 0.404; (B) *p* = 0.726]. These results indicate that the amounts of these mRNAs in the CGCs always appear in the same ratio regardless of the RT method used, with LymCREB2 and LymCBP being 10- and 2-fold higher than LymCREB1, respectively.

**Table 3:** Results of linear regression *F*-tests analyzing the correlation between the mRNA levels of LymCREB1 vs. LymCREB2 and LymCREB1 vs. LymCBP calculated from absolute quantification data. Regression lines are shown in Fig 7. DFn: degrees of freedom in the numerator, DFd: degrees of freedom in the denominator.

	*n*	Slope	*F* value	DFn	DFd	*p* value	Significance
**LymCREB1 vs. LymCREB2**							
RT method 1	8	11.80 ± 1.60	54.71	1	6	3.14E-04	significant
RT method 2	8	9.50 ± 0.83	131.2	1	6	2.66E-05	significant
**LymCREB1 vs. LymCBP**							
RT method 1	8	1.80 ± 0.63	8.08	1	6	0.029	significant
RT method 2	8	2.65 ± 0.66	16.21	1	6	0.0069	significant

## Discussion

In the present study, we trained snails using a CTA training protocol and investigated changes in the mRNA levels of LymCREB1, LymCREB2, and LymCBP in single-isolated CGC using different qPCR methods. Our findings indicated that the choice of RT and qPCR procedures affects the mRNA quantification results.

In this study, mRNA levels at the single-cell level were determined in the same cDNA samples by relative quantification using the ΔΔCt method with a fluorescent dye and absolute quantification using a standard curve with a TaqMan probe. Compared with the results of the relative quantification method (Fig 4A, 4D and 4G), which revealed no significant differences between groups (Fig 4G–4I), the absolute quantification method (Fig 5B, 5E and 5H) showed that the LymCBP mRNA level was significantly higher in the naïve-control than in the BW-trained snails (Fig 5H). The graph patterns, however, were almost the same for LymCREB1, LymCREB2, and LymCBP normalized using 3 different methods (18S/GAPDH, 18S only, and GAPDH only). Although Ståhlberg and Kubista mentioned that “reference gene normalization is not applicable to single-cell data” [42], relative quantification seems to be useful for measuring LymCREB1, LymCREB2, and LymCBP. Relative quantification has also been adopted for determining the mRNA levels of CREB1 and CREB2 in single neurons of another molluscan species *Aplysia* using its 18S ribosomal RNA as a single reference gene [43]. As Schefe *et al*. have pointed out, the assumption of 100% PCR efficiency may lead to significant errors in gene-specific relative expression ratios obtained from real-time PCR analysis [15]. The formula of “gene expression’s Ct difference” introduced by Schefe *et al*. would compensate for these drawbacks. It would be more economic than the standard-curve method and ensures a higher accuracy in determining gene expression ratio than the ΔΔCt method and the standard-curve method. Although we agree with these claims, the use of their formula requires high skill, so we would like to leave the use of the method by Schefe *et al*. for a future issue.

A possible reason for the differences in the absolute quantification results in the present study is the difference in the RT method as Ståhlberg et al. mentioned that experimental variations in qPCR are mainly attributable to the reverse transcription steps [44]. Ståhlberg and colleagues quantified 5 genes that were reverse-transcribed using random hexamer, oligo d(T), single gene-specific primers, and a mixture of gene-specific primers, and concluded that the optimal RT priming strategy depends on the gene [44]. The use of random hexamer and oligo d(T) primers gave the highest yields for 3 genes [β-tubulin, caveolin 1D (CaVD1), and GAPDH] and 2 genes [insulin II and glucose transporter 2 (GluT2)], respectively, while no gene-specific primers were suitable for any of these genes. Here, by comparing the absolute quantification results using samples prepared by RT methods 1 and 2 (Fig 5B–5I), the mRNA levels in samples processed using RT method 2 were calculated to be up to 3.3-times higher than those in the in samples processed using RT method 2. The RT method 1 samples were reverse-transcribed with a mixture of random primers and oligo d(T) primers, while a mixture of the gene-specific RT primers was used for the RT method 2 samples. To measure LymCREB1, LymCREB2, and LymCBP, gene-specific primers might give higher yields and thus be suitable. Another possible factor contributing to the variations in our results is reverse transcriptase. For the 2 RT methods, we used different enzymes although they were derived from the same origin, the reverse transcriptase of M-MLV (Moloney murine leukemia virus). Future studies should examine the differences in yields produced by these enzymes.

We previously published reports of the LymCREB1 copy numbers in individual isolated CGCs [17] and in the whole cerebral ganglia [45], which contain the CGCs. In particular, in the isolated CGC, the LymCREB1 mRNA copy number was below the limit of quantification (< 25 copies) and could not be determined. In the present study, however, the average LymCREB1 mRNA copy number was approximately 4500 using RT method 1 and approximately 15,000 using RT method 2 (Fig 5B and 5C). Furthermore, the LymCREB2 mRNA copy number was previously estimated to be 30–240 copies in a single CGC [17], but in the present study, RT method 1 detected 40,000 copies and RT method 2 detected 125,000 copies (Fig 5E and 5F). That means that more than 1000 times more mRNA was detected in the present study. The difference in the method between the present study and the previous experiment was the choice of the same *Taq* enzyme used for PCR. However, the status of this enzyme when purchased from the company were different between previous and present experiments. In previous experiments, we used AmpliTaq Gold DNA polymerase (Applied Biosystems) supplied separately from the buffer and blended dNTPs. TaqMan® Gene Expression Master Mix (Applied Biosystems), which was used in the present study, is a premixed solution containing AmpliTaq Gold DNA Polymerase, buffer, and dNTPs. Difference of compositions of the buffer prepared for premade master mix and provided separately from *Taq* polymerase might be an essential factor for the difference of qRT-PCR results. However, the company does not indicate the detail of the buffer composition.

Our previous single-cell qPCR study using CGCs showed that the LymCREB2 mRNA copy number is approximately 10-fold higher than the LymCREB1 mRNA copy number [17]. In the present study, absolute quantification using different RT methods also showed that the LymCREB2 mRNA copy number was 10-fold higher than the LymCREB1 mRNA copy number in naïve-control snails regardless of the RT method used (Fig 7A), suggesting that the expression ratio of LymCREB2/LymCREB1 in the CGCs is constantly maintained at around 10-fold in snails. CREB2 is reported to act as a repressor of CREB1 by forming a heterodimer [39, 40], suggesting that LymCREB2 regulates that learning does not easily occurs by inhibiting the function of LymCREB1. If snails frequently acquire the conditioned taste aversion, kinds of food that snails can eat are extremely limited. In terms of survival strength, it might be advantageous not to learn so easily. Similarly, the LymCBP/LymCREB1 ratio calculated from the absolute quantification data showed that the LymCBP mRNA copy number is about twice that of the LymCREB1 mRNA copy number (Fig 7B). LymCREB1 is suggested to interact with both LymCREB2 and LymCBP, and the LymCREB1 mRNA level was the lowest among the 3 genes in the CGCs. In summary, for accurate measurements of mRNA expression in single cells, it is important to consider differences in the reverse-transcription protocols and between the absolute and relative quantification of qPCR.

## Supporting information

S1 Fig Typical views of agarose gel electrophoresis of PCR products amplified by (A) relative and (B) absolute quantification showing the primer efficacy and specificity. The total RNA was purified from the central nervous systems (CNSs) of unconditioned snails. The reverse transcription was performed by RT Method 1 and 2 for the panel (A) and (B), respectively. PCR was performed in duplicate, and each sample was separated with a 2% agarose gel. The single bands of the PCR products of LymCREB1 (67 bp), LymCREB2 (142 bp) and LymCBP (63 bp) were observed at the length as expected, showing that the primer sets were enough efficient and specific. The arrowheads show the wells of the agarose gel.
(TIF)

S1 File All raw data as supplemental information, containing (1) scores of condition, (2) relative quantification Ct value, and (3) absolute quantification value. (XLSX)
