# Supplementary figures and images for: Comparison between relative and absolute quantitative real-time PCR applied to single-cell analyses: Transcriptional levels in a key neuron for long-term memory in the pond snail
Source: PLoS One. 2022 Dec 12;17(12):e0279017. doi: 10.1371/journal.pone.0279017 (PMC9744327; doi:10.1371/journal.pone.0279017)

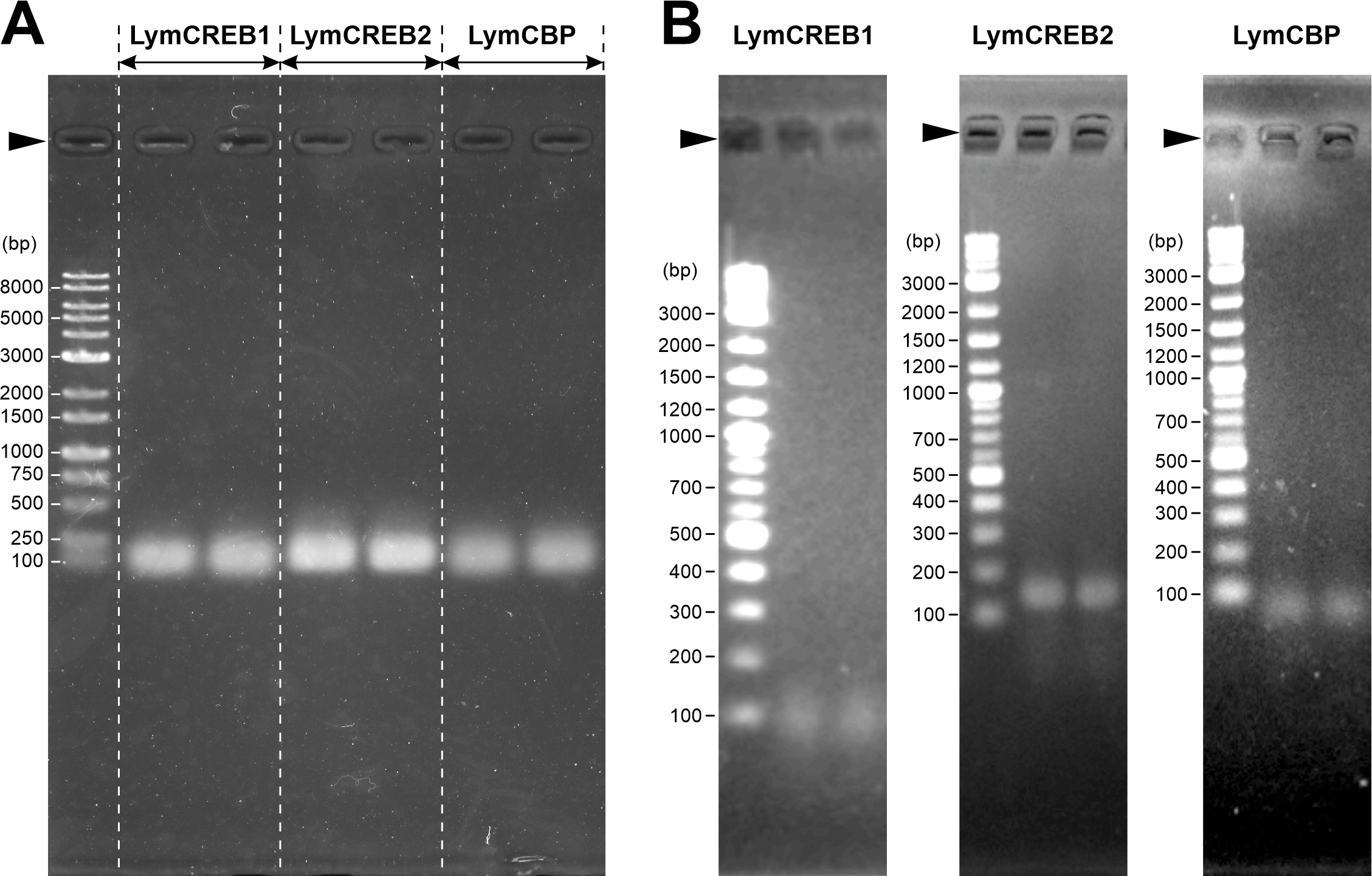

Supplement: S1 Fig — Typical views of agarose gel electrophoresis of PCR products amplified by (A) relative and (B) absolute quantification showing the primer efficacy and specificity. The total RNA was purified from the central nervous systems (CNSs) of unconditioned snails. The reverse transcription was performed by RT Method 1 and 2 for the panel (A) and (B), respectively. PCR was performed in duplicate, and each sample was separated with a 2% agarose gel. The single bands of the PCR products of LymCREB1 (67 bp), LymCREB2 (142 bp) and LymCBP (63 bp) were observed at the length as expected, showing that the primer sets were enough efficient and specific. The arrowheads show the wells of the agarose gel. (TIF) [file pone.0279017.s001.tif]
